# Significant protection from infection and AIDS progression after gastrointestinal and oral vaccinations, respectively, with a SIV DNA/rMVA vaccine

**DOI:** 10.1186/1742-4690-9-S2-O1

**Published:** 2012-09-13

**Authors:** A Aldovini, M Manrique, A Cobo-Molinos, P Kozlowski, A Carvlle

**Affiliations:** 1 Children's Hospital Boston, Harvard Medical School, Boston, MA, USA; 2 Louisiana State University Health Sciences Center, New Orleans, LA, USA; 3 New England Primate Research Center, Harvard Medical School, Southborough, MA, USA

## Background

Nasal SIV vaccination can significantly protect from AIDS progression.

## Methods

We compared four mucosal routes of vaccination in four groups of seven female Rhesus Macaques (RM) each, immunized in the oral cavity (O), gastrointestinally (GI), nasally (N) and vaginally (V) with mutated proviral SIV, IL-2 and IL-15 DNAs and SIV rMVA. Vaccinated and control animals were challenged vaginally with repeated low-dose of SIVmac251.

## Results

Only N vaccination induced a significant increase in plasma SIV-IgG titers. Significantly higher systemic, rectal and vaginal SIV-specific T-cell responses were detected in the oral group during the immunization. The median number of challenges required to become infected was significantly higher in the GI group (32; 16 for O, 12 for V, 9 for N, 11 for controls). Repeated SIV exposure expanded vaginal anti-SIV T-cells in some of the animals. Seven vaccinated RM (3 in the O, 3 in the N and 1 in the GI group) suppressed the viremia after the initial infection peak and maintained it undetectable over the course of the trial. Immunized, infected animals had significantly lower levels of systemic T-cell immune activation, better preservation of CD4+ central memory and α4β7high+ CD4+ T-cells, with consequent better protection from AIDS. However a lower protection from AIDS progression was observed in the GI group compared to the other vaccinated RM, with a median survival of 24 weeks. A significantly higher loss of CD4+ CM T-cells, detected early on in this group, correctly predicted its poor long-term outcome.

## Conclusion

Protection from infection in The GI group correlated with higher anti-SIV CD8+ T cells responses in vaginal T-cells on the day of first challenge. More than 50% of the O and N vaccinated RM were still disease-free 72 weeks after infection, and protection correlated with levels of systemic anti-SIV IFN-g+/CD8+ T-cells on the day of first challenge.

